# Specific mating behavior of Malayan pangolin (*Manis javanica*) in captivity

**DOI:** 10.1038/s41598-023-35391-2

**Published:** 2023-05-26

**Authors:** Dingyu Yan, Xiaobing Guo, Xiangyan Zeng, Miaomiao Jia, Li Tao, Xiaoting Wang, Lun He, Mingzhe Li, Zhiming Guo, Shanghua Xu, Baocai Li, Peng Zeng, Shousheng Li, Yongjie Wei

**Affiliations:** 1 Guangxi Forestry Research Institute, Nanning, 530002 Guangxi People’s Republic of China; 2 Guangxi Institute of Veterinary Research, Nanning, 530001 Guangxi People’s Republic of China; 3 China Wildlife Conservation Association, Beijing, 100714 People’s Republic of China; 4 Guangxi Terrestrial Wildlife Rescue Research and Epidemic Disease Monitoring Centre, Nanning, 530003 Guangxi People’s Republic of China

**Keywords:** Animal behaviour, Behavioural ecology

## Abstract

Pangolin is a mysterious animal in the Family Pholidota, Mammalia. Malayan pangolin (*Manis javanica*) is one of eight existing species and is listed in *Manis*. With the number of wild pangolins (*Manis* spp.) rapidly decreasing, captive breeding has become an important way to protect them from extinction. The research on mating behavior of pangolins is an important content to understand its reproductive characteristics and develop breeding management. From 2016 to 2022, a total of 360 mating events were observed in six males and 24 females through closed circuit television (CCTV) surveillance. The results show that males do not engage in complex courtship behavior before mating. In addition, we found that male pangolins adopted a ventrolateral mating position. Once males selected the side (left/right) of the female pangolin from which to approach to mate, they usually remained on the same side for subsequent mating, suggesting that male pangolins may have a preference in mating position. Finally, all mating events were observed at 1.72 ± 1.47 (n = 83, Mean ± SD) days after cohabitation and adjustment time before mating (from the male touching the female to intromission) took 4.98 ± 3.86 mins (n = 323). During mating, males hugged females and remained still for 47.37 ± 10.08 seconds (n = 323), which is the ejaculation and post-ejaculation quiescent time. Remarkably, we observed for the first time two peak mating times, 19:00 to 22:00 and 1:00 to 3:00, suggesting that they may have a preference for mating times. This study provides new insight into the mating behavior of *M. javanica* and contributes to the development of scientific conservation measures to improve the reproductive capacity of *M. javanica*.

## Introduction

Pangolins are native to tropical and subtropical Asia and sub-Saharan Africa, with eight extant species recognized^1,2^. Of the eight extant pangolin species, Malayan pangolin (*Manis javanica*), Chinese pangolin (*M. pentadactyla*) and Palawan pangolin (*M. culionensis*) are critically endangered^3–5^, and the former inhabits Southeast Asia and southern Yunnan Province in China^4,6^. Overhunting and habitat destruction have resulted in a dramatic decline of wild pangolin populations worldwide^4,7^; in fact, all eight pangolin species were upgraded from Appendix II to I of the Convention on International Trade in Endangered Species of Wild Fauna and Flora in 2016. As pangolin conservation has become more and more of a concern, artificial breeding is also receiving more attention as a major measure for ex situ conservation of wild pangolins. Although pangolins have been maintained in captivity for more than 160 years, breeding pangolins in captivity remains a challenge^8^. It is well known that pangolins have a weak immune system in captivity^9–11^, so,improving the rehabilitation and reproduction techniques of rescued pangolins is of great significance for maintaining artificial populations and preventing pangolins from going extinct. Many previous studies have been conducted on the behavior of *M. javanica*, including studies of the allocation and regularity of activities under mixed-conservation conditions^12^, behavioral observations under captive conditions^13^, their behavioral patterns and time budget^14^, as well as the home range, activity cycles, and natal den usage of female *M. javanica*^15^. The copulatory behavior of pangolins has also been reported, for example van Ee (1966) observed mating in Temminck’s pangolin (*Smutsia temminckii*), in which the male mounted the female sideways^16^. In addition, Yu et al. (2016) observed that *M. javanica* adopts a ventral-to-ventral posture for mating with limbs tightly adpressed during copulation^17^ and Zhang et al. (2020) also studied the mating behavior of two *M. javanica* during a five-day breeding period and found that their mating position was lateral-ventral and classified the mating system as the 9th or 11th pattern under both Dewsbury’s and Dixson’s classification systems respectively^18^. However, these studies used very small sample sizes and short observation periods. Therefore, the observations might not be representative and accurate. Here, we systematically analyzed and summarize six years of mating behaviour of the captive and critically endangered *M. javanica*, in order to better understand mating behaviors and how this may in fluence their reproduction.

## Results

### Definition and decription of the six terms related to mating behavior

We summarized six terms related to mating behavior, namely Mating position, Mating willingness, Mating avoidance, Enforced mating, Adjustment time before mating, Ejaculation and post-ejaculation quiescent time. At the same time, six technical terms are described (Table 1).

**Table 1:** Definition and decription of the six terms related to mating behavior.

Definition	Decription
Mating position	The male decides which side of the female's body to mate from, such as side-riding, back-riding, etc
Mating willingness	Males usually actively follow and mount the females. If the female is willing to mate, she will stop and receive the mating
Mating avoidance	If the female is not willing to mate or is afraid, she will hide. There are two ways for females to hide from males: some flee into their dens, while others block the entrance of their dens with their backs
Enforced mating	Sometimes, if a female pangolin has blocked the den entrance, the male will pull her out using his front limbs. Once the male has successfully dragged part of the female out of the den, they can mate
Adjustment time before mating	From the male touching the female to intromission
Ejaculation and post-ejaculation quiescent time	During mating, males hugged females and remained still

### Pre-mating behavior

Before mating, male pangolins have no complex courtship behavior (Supplementary Videos 1 & 2). Males usually actively follow and mount the females. If the female is willing to mate, she will stop and receive the mating. In contrast, if the female is not willing to mate or is afraid, she will hide. There are two ways for females to hide from males: some flee into their dens, while others block the entrance of their dens with their backs. Sometimes, if a female pangolin has blocked the den entrance, the male will pull her out using his front limbs. Once the male has successfully dragged part of the female out of the den, they can mate.

### Mating process

**Figure 1:**
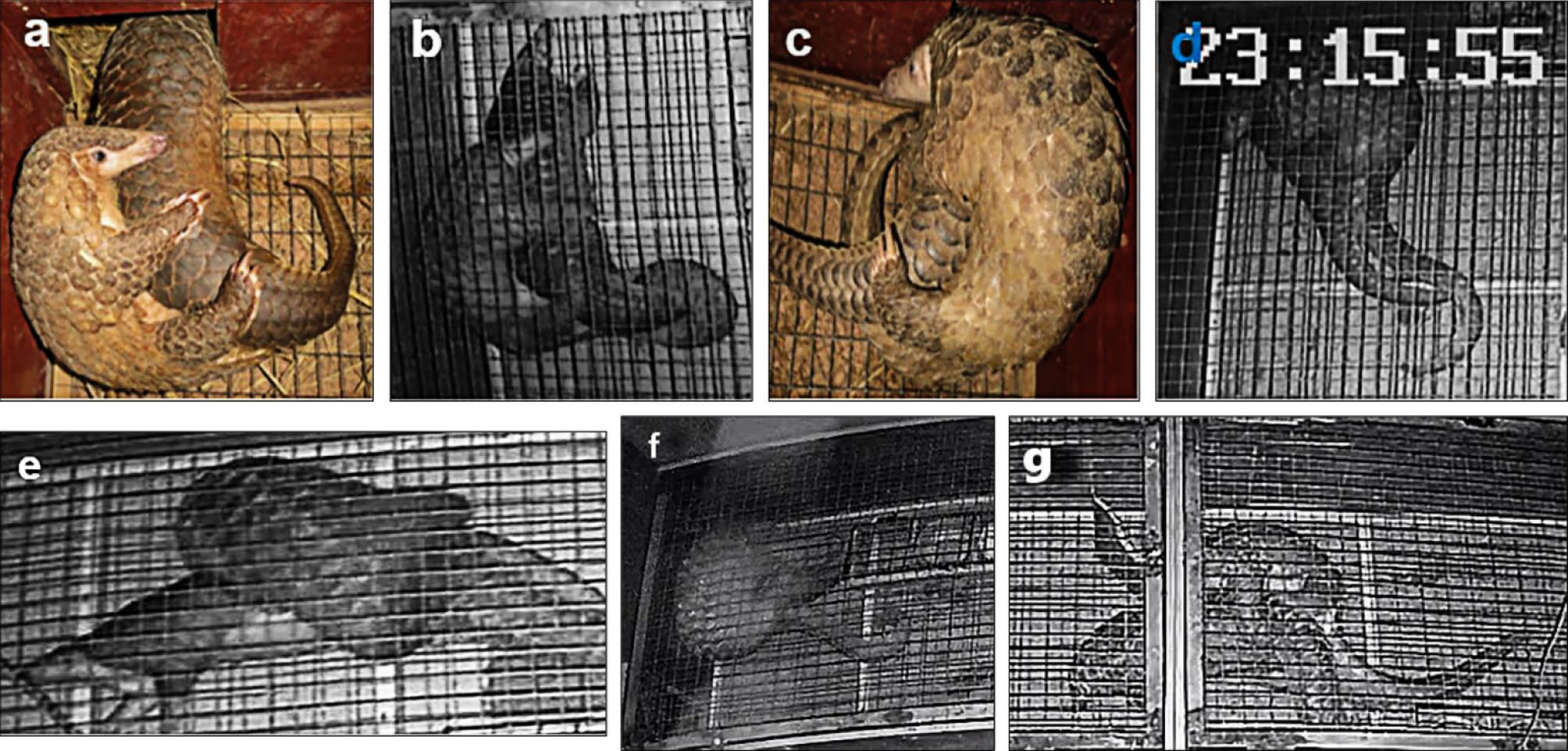
Different mating postures observed in captive *M. javanica*. (**a**) WM9 mating with WF14 from her left side (2017/11/19), (**b**) WM6 mating with WF12 from her left side (2017/08/24), (**c**) WM12 mating with WF2 from her right side (2017/04/17), (**d**) WM8 mating with WF10 from her right side (2017/07/22), (**e**) WM6 mating with WF5 from her left side (2018/01/08), (**f**) SG7 mating with WF6 from left side (2021/05/26), (**g**) SG14 mating with FG24 from her right side (2022/02/04) (Photos: Dingyu Yan).

When a male and a female were randomly selected to mate, we opened the door that connects the two active areas. During mating, the males usually mounted onto the back of the females and held their abdomens. After that, the males moved their forelimbs to the females’ sides, with most of the females lying down on their stomach or standing while a few lay on their side. The male copulates in a “side-ride” position by bending his body and bringing his genitals close to the female. On three occasions out of 323 observations, the female was overturned by the male and laid down on her side. When this happened, the male would stand on his hind legs, bend over, and move the female onto his preferred side prior to initiating mating (Fig. 1e). Male pangolins took 4.98 ± 3.86 mins (n = 323) to adjust their positions before intromission (Supplementary Table 4). For example, when WM6 mated with WF5, the adjustment time was about 4.3 mins (22:21:00 to 22:25:20) (Supplementary Video 1); When WM6 mated with first filial generation female FG10, the adjustment time was adjusted for 10 mins (20:42:10 to 20:52:30) (Supplementary Video 2). During the mating period, the males held their female partners tightly and had a quiescent time (the period from ejaculation to the time that penetration ceased) of 47.37 ± 10.08 seconds (n = 323) (Supplementary Table 4). For instance, WM6 mated with WF5 with a relative quiescent time from 22:25:20 to 22:26:05, lasting 45 s (Supplementary Video 1); WM6 mated with the fist filial generation female FG10 with a relative quiescent time from 20:52:30 to 20:53:20, lasting 50 s (Supplementary Video 2). When mating was completed, the male released the female's abdomen and would not chase her anymore, which was taken to indicate the completion of mating. Interestingly, if the approximately 45-s quiescent time was not observed, the male would not proactively release the female and would continue to chase her, indicating that the mating process was not completed. Both sexes would not sniff each other and no olfactory behavior nor cunnilingus (the act of touching a female’s vulva with the tongue) was observed.

### Male pangolins show a preference in mating position

Our data suggest that males mainly adopt the "side-riding" or "belly-side riding" position for mating, and that males also decide which side to mate with the females (Supplementary Videos 1 & 2). Interestingly, once a male had previously chosen the mating side, they would retain the same position for most or all of their future mating, suggesting that male pangolins may have a preference to mating position (Supplementary Table 2). For example, WM6 had 139 mating events with 12 females and WM9 had 53 mating events with 10 females, with both males consistently mating with females from the left side (Supplementary Table 2, Fig. 1a, b, e). WM8 mated 57 times with nine females. Of these matings, 56 occasions were from the female’s right side and only one instance was from the left side. WM12 mated 34 times with one female, with all matings occurring from the right side of the female (Supplementary Table 2, Fig. 1c, d). SG7 mated 62 times with ten females and SG14 mated 15 times with one female (Supplementary Table 2, Fig. 1f, g).

### Pangolins show a preference in mating time

**Figure 2:**
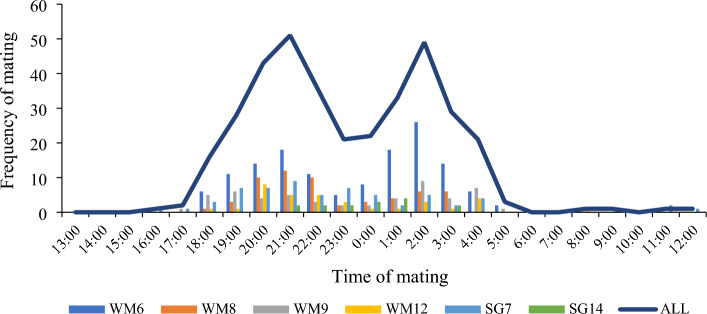
Bias of mating time distribution of six male *M. javanica* over 24 h. Based on a large number of mating frequency statistics, the distribution of male mating times is described, as well as the male's preferred mating time in a day.

Based on our 360 observed mating events from October 2016 to September 2022, most mating events occurred between 18:00 and 5:00 (97.8% of total matings), with two clear peak times from 19:00 to 22:00 and 1:00 to 3:00, suggesting that these may be the preferred times for pangolins to copulate (Fig. 2).

### Pangolin mating is aseasonal

**Figure 3:**
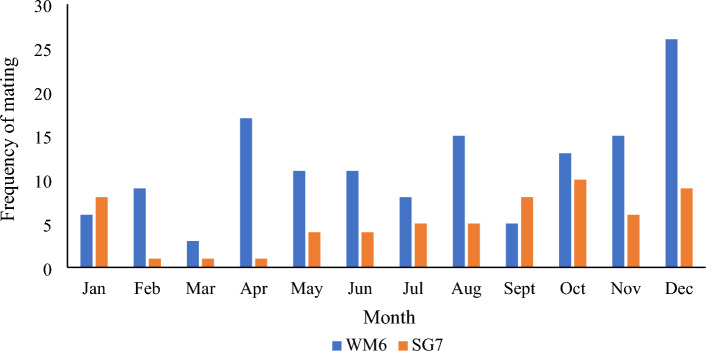
Distribution of mating time of two male *M. javanica* over the course of a year. Based on a year's mating records of two male pangolins.

**Figure 4:**
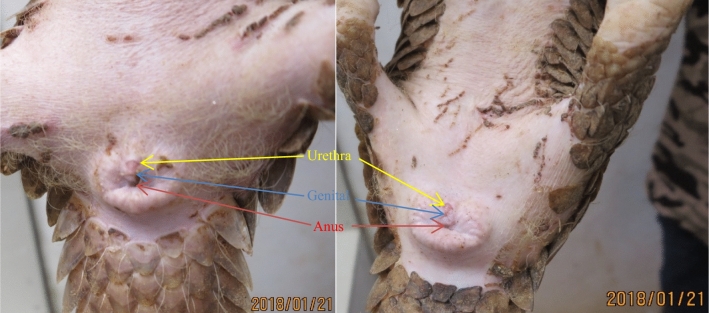
Genital shape of FG10 and WF5 1–2 days after mating. FG10: an eight-month-old female cub; WF5: FG10's mother.

**Figure 5:**
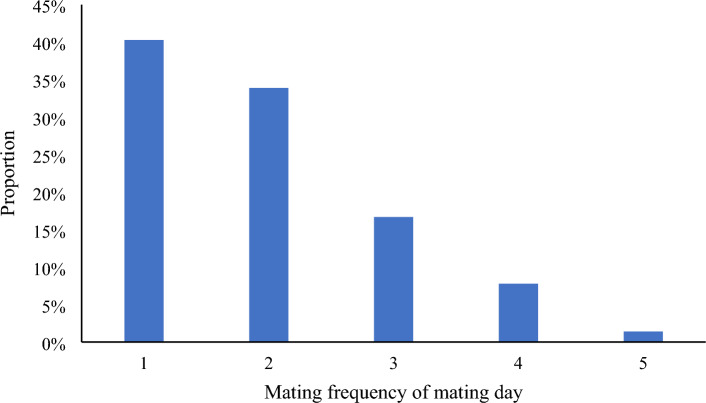
Proportion of mating frequency of captive *M. javanica* during mating days. 1–5 refer to the number of completed matings within 24 h of the occurrence and completion of mating. E.g. a total of 500 matings are recorded, and the number of matings completed once in one day is 200 times, and the ratio is 40%, and 200 mating days are required; 2 matings are completed in one day, and 200 matings are recorded, and the ratio is 40%, which requires 100 mating days.

To examine whether pangolins display seasonal mating behavior, we analyzed the mating time distribution of two male pangolins (WM6 and SG7) (Fig. 3). The results showed that mating was random and aseasonal. We also performed an analysis of their mating time. Since female pangolins do not have estrus features for example, the genitals of WF5 and FG10 barely change after mating (Fig. 4). Therefore, they were not selected for mating based on any signs of estrus. Instead, each pangolin pair was selected randomly for mating. Our data showed almost all females began to mate within five days of cohabitation, with most of the mating occurring on the first and second days of cohabitation. Pangolins mated 1–5 times per day (average 1.5; Fig. 5), with 1–2 matings per day accounting for 74.17% of the total number of mating.

### Correlation of mating behavior parameters in pangolins

In order to explore the relationship between mating behavior parameters, SPSS v.19.0 was used to analyze the pairwise correlations between Average number of days between cohabitation and mating, Number of copulations, Number of litters produced and Mean mass needed to mate normally. The results show that there was no correlation among the four mating behaviour parameters. (Supplementary Table 3).

## Discussion

Strengthening pangolin protection is the consensus of all governments where pangolins are distributed. Captive breeding of pangolins confiscated from the illegal trade to establish a sustainable artificial population is one of the most effective ways to protect the species from extinction. Pangolin breeding technology is undoubtedly one of the most critical links. Only by mastering the methods of normal breeding of the species in captivity can we improve the reproduction rate of pangolins and better protect them. Here we report on a long-term and comprehensive survey of the sexual behavior of the critically endangered *M. javanica*. In this study, the mating behavior was summarized and the results showed that the mating could be completed 1.72 ± 1.47 days after random cohabitation. There is no complex courtship behavior before mating and the mating process is relatively simple, which mainly depends on the male initiative, and there is no genital licking behavior after mating. Females have no obvious estrus characteristics during mating (Fig. 4), and the females can mate with males at any time, even during pregnancy. In addition, our published paper summarizing the mating effect shows that about 50% of females mated during pregnancy. Some non-pregnant females get pregnant up to 80% during their first cohabitation and copulation. Therefore, it is a special and effective reproductive strategy for females of this species to mate at any time and get pregnant. This makes up for the species' low mobility, isolation, solitary nature, unusual feeding habits, and the fact that most females produce one offspring per litter.

The most typical mating method in mammals is the “ventral-to-dorsal" style^19^. Consistent with the observations in previous studies, we found that *M. javanica* adopts the “male mounts the female sideways” mating posture ^16,20^. We believe that having a preferred side from which to initiate mating may help male pangolins align their penis. Once a male has selected a preferred side from which to initiate mating, they usually select the same side in future matings. This is true of the wild generation, as well as the filial generation males bred in the center. Moreover, even if the tailless M9 (tail surgically removed due to infection) remains on the same side, this male fixed-side mating behavior is interesting and rare. In fact, it’s very difficult for pangolins to mate successfully. Males took longer (4.98 ± 3.86 min) to adjust from touching females to penetration. There are three main reasons for this. First, both males and females have broad tails. Second, the genital of the female is close to the anus, which is surrounded by protruding glands and the anus is in the middle of the glands. Third, there is no swelling or evagination of the female genitalia during mating, as in pigs^21^, dogs^22^, and civets^23^. Our data suggest that pangolins do not exhibit a strictly seasonal mating, as mating was recorded during every month. This is consistent with previous studies showing no seasonality in reproduction ^24–26^.

Previous studies applied the Dewsbury system and the Dixson system to define the mating patterns of captive civets and Mongolian wild donkeys in nature reserves^27,28^. The mating pattern of *M. javanica* conforms to the 15th type in the Dewsbury system, showing no mechanical ties between the genitals, no pelvic thrusting following penetration, a single intromission event and multiple ejaculation events^29^. Since male pangolins mated for less than a minute, their mating patterns are also assigned to the 16th type in Dixson system, showing no lock, no tics, single intromission event, and short holding time^30^. Zhang et al. (2020) also studied the mating behavior of the Javan buttercup and classified the mating system as the 9th or 11th mode under the Dewsbury and Dixson classification systems, respectively^17^ . However, these studies only used one male and one female in their sample, and the observation period was only 5 days. Given the large amount of data we recorded on mating events, there may be better confidence in our results.

Strikingly, we are the first to report two preferred peak times (19:00–22:00 and 1:00–3:00) for pangolins to copulate. All pangolins mated between 17:00 and 08:00, but the mating frequency was highest during these two peaks. In particular, each male mating basically follows two peak time patterns (Fig. 2). This is an interesting phenomenon, and why there is such a phenomenon needs more research. In addition, our finding is slightly different from a previous study, which found that all *M. javanica* mated between 20:00 and 08:00^18^. Due to the large sample size of this study (360 mating events), the results of this study have a better resolution when analyzing the preferred times of pangolin copulation.

Through research, we have obtained the behavioral rules of pangolin mating, and conclude that captive pangolins have no obvious estrus characteristics or strict estrus period. Randomly cohabitated females were mated, some even during pregnancy. The mating process is simple and the mating time is short, but with a long mating preparation time, and there is a preferred peak mating time. Therefore, in the breeding management of pangolins, as long as males are willing to mate, the time of cohabitation with females can be reduced to less than 5 days, which can improve the mating efficiency of males who are willing to mate. It is recommended that females remain cohabited with males for 1–2 months after their first mating to increase their conception rate^26^. And, in order to observe mating behavior without blind spots, we designed the den so that mating cannot be done inside the den. The size of the den in winter and summer is 40 × 35 × 28 cm, and the length of the male head and tail is 85–110 cm and that of the female is 70–90 cm. Moreover, the pangolin's tail is wide enough for mating to be impossible. We designed an activity field outside the den (cage, 120 × 80 × 50 cm), the cage is not covered, to ensure that video monitoring is not affected, and breeding behavior can be recorded. We have also designed indoor and outdoor fences imitating the wild, which have not yet been put into use, which might greatly restore the wild behavior and improve the efficiency of successful mating. What’s more, the results also show that females can mate and conceive during pregnancy, so it is suggested that a female can be artificially manipulated to mate with different males to increase the conception rate. Males and females are mixed in captivity, and the feeding time should avoid the peak mating time so as to reduce interference, thereby improving the success rate of mating, and provide an important basis for the breeding management of pangolins. Meanwhile, it is unclear whether captive and wild pangolins share similar mating behavioral characteristics. Therefore, replicating this study in wild pangolin populations to further confirm their mating patterns may provide a reference for the study of wild pangolin breeding behavior. Finally, the results of this study indicate that there is no correlation between mating behaviour parameters and further study is needed.

## Methods

### Ethics statement

This project was approved by the Forestry Department of Guangxi Zhuang Autonomous Region, Guangxi Province (domestication and breeding permit of national key protected wild animals; Permit Number: A2016008). All works and protocols were approved by the Biology Ethics Committee of the Guangxi Forestry Research Institute [Reference Number: GXFI (A2016006)]. All methods were carried out in accordance with relevant guidelines and ARRIVE guidelines.

### Experimental animals

The experimental animals in this study were six male and 24 female *M. javanica*. Among them, 14 females and four males were confiscated from smugglers by law enforcement officers between September 2013 and April 2016, while 12 females and two males (SG7 and SG14) were born in captivity between May 2016 and July 2020 (Supplementary Table 1).

### Feeding and nursing conditions

The study was conducted at the “Pangolin Rescue and Breeding Centre” (PRBC) of Guangxi Forestry Research Institute, Nanning, China. PRBC is located at latitude 22.9° north, with temperatures in January (the coldest month) averaging 12.8 °C. The housing, husbandry and care for Malayan pangolins at PRBC have been described in our previous studies^26^. As *M. javanica* inhabit tropical environments, we kept them in indoor cages which consisted of three areas: an activity area, an insulated wooden den for winter and an underground den for summer. Each pangolin was kept in a single cage, with an activity area size of 120 cm × 80 cm × 50 cm. Each cage was equipped with wire mesh (2.5 cm × 5.0 cm) that helped to keep the cage clean. The small wooden winter den (40 cm × 35 cm × 28 cm) was equipped with a thermostat, and the dimension of the underground summer den was about 40 cm × 35 cm × 28 cm. And, in order to observe mating behavior without blind spots, we designed the den so that mating cannot be done inside the den. The size of the den in winter and summer is 40 × 35 × 28 cm, and the length of the male head and tail is 85–110 cm and that of the female is 70–90 cm. Moreover, the pangolin's tail is wide enough for mating to be impossible. We designed an activity field outside the den (cage, 120 × 80 × 50 cm), the cage is not covered, to ensure that video monitoring is not affected, and breeding behavior can be recorded. The indoor temperature was not regulated, except the temperature of the insulated winter den which was maintained at 24–26 ℃ during winter. These settings allowed pangolins to select which den to use according to different seasons and indoor temperatures. For instance, they prefer to sleep in the insulated winter den when the indoor temperature is low (e.g., < 23 °C) but prefer the underground summer den when the indoor temperature is high (e.g., > 25 °C). To minimize the risk of infections, all cages were cleaned monthly and the dens were disinfected with a gas torch and all pangolins were fed an artificial diet consisting of black ant powder, silkworm pupae powder, mealworm powder, soy protein powder, termite mound mud and a small amount of vitamin complex^26^. The food was mixed with water and then blended into a fluid. Pangolins were fed 250 to 400 mL of food once a day during the period 17:00–20:00. Clean water was provided in a separate bowl. All foods are stored in a dry, hygienic environment to minimize the growth of pathogens. At the end of the project, all live pangolins will remain at our centre for long-term research.

### Monitoring methods

From October 2016 to September 2022, wide-angle infrared closed-circuit television (CCTV) cameras were used to observe the mating behavior of *M. javanica* during both day and night. Since the estrus mechanism of female pangolins remains unknown, the female pangolins were not selected for mating based on signs of estrus. Instead, we periodically selected one male and one female pangolin at random and housed them in adjacent cages for mating. The doors of these adjacent cages were opened and the infrared camera continuously monitored both cages in their entirety in real-time. Mating date, time, and mating method were determined by video playback. Some of the replays were not recorded due to equipment resons, so there were 323 detailed records of mating, and the remaining 37 recorded only dates of mating. Typical posture of mating helped to determine whether a male ejaculated or not and whether he finished mating (Fig. 1). The peak interval to mating and diel interval for mating was determined after considering all mating events. Completion of ejaculation was determined based on the observed mating actions. Any changes in the male’s mating behavior was recorded while mating with the same or a different female.

### Statistical analysis

A total of 360 mating events were recorded from October 2016 to September 2022. The recorded information mainly includes the ID of the mating individual, the mating date, the mating time and the subsequent production. The mean and standard deviation of all observations (n = 360) were calculated using the software package SPSS v. 19.0 (IBM, Armonk, NY, USA). The mating information table and related analysis chart were drawn by Microsoft Office Excel 2016.

## Supplementary Information

Supplementary Legends.

Supplementary Tables.

Supplementary Video 1.

Supplementary Video 2.

## Data Availability

All data generated or analysed during this study are included in this published article [and its supplementary information files].
