# Supplementary material for: Specific mating behavior of Malayan pangolin (Manis javanica) in captivity
Source: Sci Rep. 2023 May 26;13:8592. doi: 10.1038/s41598-023-35391-2 (PMC10220066; doi:10.1038/s41598-023-35391-2)
Supplement: Supplementary file 1 — Supplementary Legends. [file 41598_2023_35391_MOESM1_ESM.docx]

**Supplementary Information**

**Specific mating behavior of Malayan pangolin (*Manis javanica*) in captivity**

**Dingyu Yan**^1,🖂,+^ **Xiaobing Guo**^1,+^, **Xiangyan Zeng**^1^, **Miaomiao Jia**^1^, **Li Tao**^2^, **Xiaoting Wang**^3^, **Lun He**^3^, **Mingzhe Li**^3^ , **Zhiming Guo**^3^, **Shanghua Xu**^1^, **Baocai Li** ^1^, **Peng Zeng**^4^, **Shousheng Li**^4^, **Yongjie Wei**^4^

^1^Guangxi Forestry Research Institute, Nanning, Guangxi, 530002, P.R. China

^2^Guangxi Institute of Veterinary Research, Nanning, Guangxi, 530001, P.R. China

^3^China Wildlife Conservation Association, Beijing, 100714, P.R. China

^4^Guangxi Terrestrial Wildlife Rescue Research and Epidemic Disease Monitoring Centre, Nanning, Guangxi, 530003, P.R. China

^🖂^corresponding.author :Yandy6@126.com

^+^these authors contributed equally to this work

**Keywords:** Malayan pangolin; mating behavior; closed circuit television; mating position; mating time

**Supplemental Video Legends**

**Video S1.** The whole mating process of WM6 mated with WF5 captured from CCTV recording.

**Video S2.** The whole mating process of WM6 mated with first filial generation female FG10 captured from CCTV recording.
