# Supplementary material for: Specific mating behavior of Malayan pangolin (Manis javanica) in captivity
Source: Sci Rep. 2023 May 26;13:8592. doi: 10.1038/s41598-023-35391-2 (PMC10220066; doi:10.1038/s41598-023-35391-2)
Supplement: Supplementary file 2 — Supplementary Tables. [file 41598_2023_35391_MOESM2_ESM.docx]

**Supplementary Tables**

**Supplementary Table 1.** Characteristics of wild and captive-bred *M. javanica* that were used for this study.

| **Pangolin ID^1^** | **Gender** | **Date received/birth** | **Initial mass^2^ (kg)** | **Mass needed to mate normally^3^ (kg)** |
| --- | --- | --- | --- | --- |
| WF1 | female | 2013/09/11 | 3.3 | 5.5–6.2 |
| WF2 | female | 2014/01/10 | 2.3 | 5.3–6.0 |
| WF3 | female | 2014/01/10 | 2.6 | 4.5–5.0 |
| WF5 | female | 2015/09/04 | 3.7 | 4.5–5.2 |
| WF6 | female | 2015/09/04 | 3.5 | 4.8–5.5 |
| WF8 | female | 2015/12/15 | 4.0 | 5.1–6.1 |
| WF10 | female | 2015/12/15 | 3.6 | 4.0–4.5 |
| WF11 | female | 2016/01/19 | 3.7 | 4.5–5.0 |
| WF12 | female | 2016/01/19 | 3.5 | 5.5–6.1 |
| WF14 | female | 2016/04/16 | 4.3 | 4.1–4.6 |
| WF15 | female | 2016/04/16 | 4.5 | 5.3–5.8 |
| WF16 | female | 2016/04/16 | 5.3 | 5.1–5.7 |
| WM6 | male | 2015/09/16 | 5.0 | 7.0–8.3 |
| WM8 | male | 2015/12/15 | 2.3 | 7.0–8.5 |
| WM9 | male | 2015/12/15 | 4.5 | 5.0–6.0 |
| WM12 | male | 2016/01/19 | 8.9 | 8.5–9.0 |
| FG3 | female | 2016/06/05 | NA | 2.8 (2017-06-17) |
| FG4 | female | 2016/06/25 | NA | 4.9 (2017-09-24) |
| FG6 | female | 2016/08/22 | NA | 3.6 (2017-08-15) |
| FG7 | female | 2016/10/18 | NA | 4.0 (2017-09-07) |
| FG16 | female | 2018/05/19 | NA | NA |
| FG19 | male | 2018/07/13 | NA | NA |
| FG21 | female | 2018/09/25 | NA | NA |
| FG22 | female | 2018/11/12 | NA | NA |
| FG24 | female | 2019/04/22 | NA | NA |
| FG26 | female | 2018/9/25 | NA | NA |
| SG4 | female | 2018/06/06 | NA | NA |
| SG6 | female | 2018/10/06 | NA | NA |
| SG7 | male | 2019/02/12 | NA | NA |
| SG14 | male | 2020/07/01 | NA | NA |

^1^WF = Wild Female, WM = Wild Male, FG = First Generation (captive-bred), SG = Second Generation. NA = Not applicable.

^2^ Initial mass is the mass of the individual at the time that it was received at our rehabilitation centre.

^3^For captive-bred individuals, the date of first copulation is noted in brackets following the mass at the time of copulation.

**Supplementary Table 2.** Details of the mating pairs, mating dates, mating approach side and mating frequency of captive *M. javanica*.

| **Male ID** | **Female ID** | **Mating dates** | **Time from cohabitation to mating (days)** | **Mating side** | **Number of copulations** | **Outcome** |
| --- | --- | --- | --- | --- | --- | --- |
| WM6 | WF1 | 2016/12/17-2016/12/20 | 4 | Left | 4 | No conception |
| WM6 | WF1 | 2017/08/29-2017/09/01 | 2 | Left | 5 | No conception |
| WM6 | WF2 | 2016/11/10-2016/11/15 | 3 | Left | 2 | Parturition on 2017/05/19 |
| WM6 | WF2 | 2017/12/30-2018/01/06 | 1 | Left | 13 | Parturition on 2018/07/22 |
| WM6 | WF3 | 2016/11/19-2016/12/01 | 1 | Left | 7 | No conception |
| WM6 | WF3 | 2016/12/22-2016/12/28 | 3 | Left | 4 | No conception |
| WM6 | WF3 | 2017/07/14-2017/07/18 | 1 | Left | 8 | No conception |
| WM6 | WF5 | 2017/12/04-2017/12/13 | 1 | Left | 7 | Parturition on 2018/06/11 |
| WM6 | WF8 | 2016/10/10-2016/10/26 | 1 | Left | 13 | Parturition on 2017/04/15 |
| WM6 | WF8 | 2017/09/24-2017/09/28 | 2 | Left | 5 | Parturition on 2018/04/04 |
| WM6 | WF11 | 2017/03/27-2017/04/14 | 2 | Left | 11 | Death with the mother |
| WM6 | WF12 | 2017/02/09-2017/02/26 | 1 | Left | 9 | Parturition on 2017/08/12 |
| WM6 | WF12 | 2017/08/23-2017/08/25 | 1 | Left | 4 | No conception |
| WM6 | WF12 | 2017/11/05-2017/11/10 | 4 | Left | 6 | Parturition on 2018/05/19 |
| WM6 | WF14 | 2017/05/01-2017/05/10 | 2 | Left | 11 | No conception |
| WM6 | WF15 | 2017/04/19-2017/04/27 | 1 | Left | 6 | No conception |
| WM6 | WF15 | 2017/08/01-2017/08/05 | 1 | Left | 3 | No conception |
| WM6 | WF16 | 2017/01/11-2017/02/01 | 3 | Left | 6 | Parturition on 2017/07/15 |
| WM6 | FG3 | 2017/06/17-2017/06/25 | 1 | Left | 11 | No conception |
| WM6 | FG6 | 2017/08/15-2017/08/16 | 1 | Left | 4 | Parturition on 2018/02/15 |
| WM8 | WF3 | 2017/10/25-2017/10/29 | 2 | Right | 5 | No conception |
| WM8 | WF5 | 2017/2/21 | 10 | Right | 2 | Mating during pregnancy |
| WM8 | WF5 | 2017/04/04-2017/04/12 | 2 | Right | 7 | Mating during pregnancy |
| WM8 | WF6 | 2017/08/02-2017/08/14 | 3 | Right | 2 | Parturition on 2018/02/15 |
| WM8 | WF10 | 2017/05/26-2017/05/28 | 1 | Right | 4 | No conception |
| WM8 | WF10 | 2017/07/21-2017/07/24 | 1 | Right | 4 | No conception |
| WM8 | WF10 | 2017/11/13-2017/11/18 | 2 | Right | 4 | WF10 died on 2018/02/08 |
| WM8 | WF15 | 2017/05/01-2017/05/04 | 2 | Right | 3 | No conception |
| WM8 | WF15 | 2017/11/29 | 5 | Right | 1 | No conception |
| WM8 | FG3 | 2017/12/2 | 1 | Right | 1 | Parturition on 2018/06/06 |
| WM8 | FG4 | 2017/09/24-2017/09/27 | 2 | Right | 6 | Parturition on 2018/04/04 |
| WM8 | FG4 | 2017/12/23-2018/01/04 | 1 | 14 Right; 1 Left | 15 | Mating during pregnancy |
| WM8 | FG7 | 2017/09/07-2017/09/11 | 4 | Right | 3 | Parturition on 2018/03/10 |
| WM9 | WF1 | 2017/11/13-2017/11/19 | 2 | Left | 6 | No conception |
| WM9 | WF1 | 2017/11/25-2017/12/02 | 1 | Left | 7 | No conception |
| WM9 | WF3 | 2017/10/30 | 1 | Left | 1 | No pregnant |
| WM9 | WF8 | 2020/6/13 | 1 | Left | 1 |  |
| WM9 | WF14 | 2017/12/3 | 1 | Left | 2 | Parturition on 2018/05/25 |
| WM9 | WF14 | 2019/3/3 | 1 | Left | 2 | Not obtained |
| WM9 | WF16 | 2019/3/11 | 1 | Left | 2 | Not obtained |
| WM9 | FG6 | 2017/11/02-2017/11/5 | 1 | Left | 6 | Mating during pregnancy |
| WM9 | FG6 | 2018/4/11 | 1 | Left | 1 | Not obtained |
| WM9 | FG6 | 2018/06/13-2018/06/16 | 1 | Left | 2 | Not obtained |
| WM9 | FG6 | 2018/07/20-2018/07/22 | 1 | Left | 2 | Not obtained |
| WM9 | FG6 | 2018/09/13-2018/09/14 | 2 | Left | 3 | Not obtained |
| WM9 | FG19 | 2019/8/26 | 1 | Left | 1 | Not obtained |
| WM9 | FG19 | 2019/9/3-2019/9/5 | 1 | Left | 4 | Not obtained |
| WM9 | FG26 | 2019/4/13-2019/4/14 | 1 | Left | 3 | Not obtained |
| WM9 | SG4 | 2019/4/4 | 1 | Left | 3 | Not obtained |
| WM9 | SG4 | 2019/12/31 | 1 | Left | 1 | Not obtained |
| WM9 | SG4 | 2020/1/1 | 1 | Left | 1 | Not obtained |
| WM9 | SG4 | 2020/3/3-2020/3/6 | 1 | Left | 2 | Not obtained |
| WM9 | SG6 | 2019/5/26-2019/5/27 | 1 | Left | 3 | Not obtained |
| WM12 | WF2 | 2017/04/03–2017/04/17 | 2 | Right | 34 | No conception |
| SG7 | WF6 | 2020/10/10-2020/10/11 | 1 | Left | 3 | Parturition on 2021/4/18 |
| SG7 | WF6 | 2021/05/26-2021/05/28 | 1 | Left | 4 | Parturition on 2021/12/2 |
| SG7 | WF8 | 2021/8/25 | 1 | Left | 3 | Parturition on 2022/2/27 |
| SG7 | WF12 | 2021/6/28 | 2 | Left | 1 | No conception |
| SG7 | WF12 | 2022/01/26-2022/01/27 | 1 | Left | 4 | No conception |
| SG7 | WF16 | 2021/09/15-2021/09/17 | 2 | Left | 6 | Parturition on 2022/3/20 |
| SG7 | WF16 | 2020/11/10-2020/11/11 | 1 | Left | 3 | Parturition on 2021/5/16 |
| SG7 | WF16 | 2020/12/3 | 1 | Left | 1 | Mating during pregnancy |
| SG7 | FG16 | 2020/10/20 | 1 | Left | 2 | Parturition on 2021/5/6 |
| SG7 | FG16 | 2021/2/16 | 3 | Left | 1 | Mating during pregnancy |
| SG7 | FG21 | 2020/12/23–2020/12/26 | 1 | Left | 7 | Parturition on 2021/6/30 |
| SG7 | FG21 | 2022/4/15 | 1 | Left | 1 | No conception |
| SG7 | FG21 | 2022/6/5 | 1 | 1 Right ;1 Left | 2 | No conception |
| SG7 | FG22 | 2022/3/7 | 1 | Left | 1 | Parturition on 2022/9/15 |
| SG7 | FG26 | 2019/11/14 | 2 | Left | 1 | Parturition on 2020/05/19 |
| SG7 | FG26 | 2019/11/29-2019/11/30 | 1 | Left | 2 | Mating during pregnancy |
| SG7 | FG26 | 2019/12/23 | 5 | Left | 1 | No conception |
| SG7 | FG26 | 2020/1/25 | 4 | Left | 1 | No conception |
| SG7 | FG26 | 2020/6/12 | 1 | Left | 1 | Parturition on 2020/12/14 |
| SG7 | FG26 | 2020/8/13-2020/8/15 | 1 | Left | 2 | Mating during pregnancy |
| SG7 | FG26 | 2020/9/29-2020/9/30 | 1 | Left | 2 | Mating during pregnancy |
| SG7 | SG4 | 2021/10/21-2021/10/22 | 7 | Left | 5 | Parturition on 2022/04/27 |
| SG7 | SG6 | 2021/7/5-2021/7/6 | 1 | Left | 5 | Parturition on 2022/01/07 |
| SG7 | SG6 | 2022/1/20 | 1 | 1 Right; 2 Left | 3 | Death with the mother |
| SG14 | FG24 | 2022/02/04-2022/02/06 | 1 | Right | 3 | Not obtained |
| SG14 | FG24 | 2022/03/17-2022/03/18 | 1 | Right | 3 | Not obtained |
| SG14 | FG24 | 2022/04/27-2022/04/28 | 1 | 1 Right; 1 Left | 2 | Not obtained |
| SG14 | FG24 | 2022/06/3-2022/06/5 | 1 | Right | 5 | Not obtained |
| SG14 | FG24 | 2022/09/8–2022/09/9 | 1 | Right | 2 | Not obtained |

**Supplementary Table 3.** Pearson correlations between Average number of days between cohabitation and mating, Number of copulations, Number of litters produced and Mean mass needed to mate normally.

| Variables | | Average number of days between cohabitation and mating | Number of copulations | Number of litters produced | Mean mass needed to mate normally |
| --- | --- | --- | --- | --- | --- |
| Average number of days between cohabitation and mating |  |  | -0.007  (0.982) | -0.207  (0.518) | 0.061  (0.852) |
| Number of copulations |  |  |  | 0.046  (0.886) | 0.471  (0.122) |
| Number of litters produced |  |  |  |  | 0.185  (0.565) |
| Mean mass needed to mate normally |  |  |  |  |  |

*Note. The P value of significance is indicated in parentheses*

*P > 0.05 indicates no significant difference.*

**Supplementary Table 4.** Statistics of mating behavior parameters of captive *M. javanica*.

| **Individual ID** | **Adjustment time before mating (minutes)** | **Quiescent time during mating (seconds)** | **Time between cohabitation and copulation (days)** |
| --- | --- | --- | --- |
| WM6 | 5.90 ± 4.71 | 47.05 ± 8.82 | 1.80 ± 1.06 |
| WM8 | 4.19 ± 2.69 | 42.81 ± 7.50 | 2.77 ± 2.49 |
| WM9 | 4.00 ± 3.52 | 39.76 ± 6.52 | 1.10 ± 0.31 |
| WM12 | 3.93 ± 1.80 | 46.18 ± 5.65 | 2.00 ± 0.00 |
| SG7 | 4.74 ± 3.30 | 57.62 ± 11.48 | 1.75 ± 1.54 |
| SG14 | 5.17 ± 2.42 | 57.33 ± 4.84 | 1.00 ± 0.00 |
| All | 4.98 ± 3.86 | 47.37 ± 10.08 | 1.72 ± 1.47 |
